# Internet-Delivered Cognitive Behavioural Therapy for Adults with Mild to Moderate Depression and High Cardiovascular Disease Risks: A Randomised Attention-Controlled Trial

**DOI:** 10.1371/journal.pone.0059139

**Published:** 2013-03-26

**Authors:** Nicholas Glozier, Helen Christensen, Sharon Naismith, Nicole Cockayne, Liesje Donkin, Bruce Neal, Andrew Mackinnon, Ian Hickie

**Affiliations:** 1 Brain and Mind Research Institute, Sydney Medical School, University of Sydney, Sydney, New South Wales, Australia; 2 Black Dog Institute, Sydney, New South Wales, Australia; 3 The George Institute for Global Health, The University of Sydney, Sydney, New South Wales, Australia; 4 Orygen Youth Health Research Centre, University of Melbourne, Melbourne, Victoria, Australia; Linkoping University, Sweden

## Abstract

**Background and Aim:**

Mild to moderate depression is common in those with cardiovascular disease and undertreated. We aimed to evaluate the effectiveness of internet-delivered Cognitive Behaviour Therapy (iCBT) on depressive symptom severity and adherence to medical advice and lifestyle interventions in adults with mild to moderate depression and high cardiovascular disease (CVD) risks.

**Methods:**

Randomised double-blind, 12 week attention-controlled trial comparing an iCBT programme (*E-couch*) with an internet-delivered attention control health information package (*HealthWatch*, n = 282). The primary outcome was depression symptom level on the nine-item Patient Health Questionnaire (PHQ-9) (trial registration: ACTRN12610000085077).

**Results:**

487/562 (88%) participants completed the endpoint assessment. 383/562 (70%) were currently treated for cardiovascular disease and 314/562 (56%) had at least one other comorbid condition. In ITT analysis of 562 participants iCBT produced a greater decline in the mean PHQ-9 score compared to the attention control of 1.06 (95% CI: 0.23–1.89) points, with differences between the two arms increasing over the intervention period (time by treatment effect interaction p = .012). There were also larger improvements in adherence (2.16 points; 95% CI: 0.33–3.99), reductions in anxiety (0.96 points; 95% CI: 0.19–1.73), and a greater proportion engaging in beneficial physical activity (Odds Ratio 1.91, 95%CI: 1.01–3.61) in the iCBT participants but no effect upon disability, or walking time/day. There were no withdrawals due to study related adverse events.

**Conclusions:**

In people with mild to moderate depression and high levels of CVD risk factors, a freely accessible iCBT programme (http://www.ecouch.anu.edu.au) produced a small, but robust, improvement in depressive symptoms, adherence and some health behaviours.

**Trial Registration:**

Australian and New Zealand Clinical Trials Registry ACTRN12610000085077

## Introduction

In high income countries, cardiovascular disease (CVD) and depression are the two leading causes of disease burden. [1] Depression is more prevalent in people with CVD and leads to a range of poorer CVD related outcomes. [2] One important factor that may explain this worse outcome is poor adherence to CVD treatment and lifestyle recommendations. Depression is a major risk for poor adherence [3] and, if depression interventions, can also produce improvements in adherence this may translate into benefits in comorbid physical health disorder management. Globally, health systems are trying to identify interventions that can deliver improvements in depression at low cost in people with these comorbid conditions. Internet interventions (e-health) may be well suited to this purpose since they have been shown to deliver efficacious psychological intervention programmes [4], [5] for depression on a large scale in a cost effective manner. [6] They are also able to overcome geographical access and human resources issues involved in face to face therapy, [6] and do not require the prescription and regulatory monitoring requirements of medication. In the UK, the National Institute for Health and Clinical Excellence (NICE) defines such approaches as “low-intensity psychosocial interventions” [7] and sees them as part of a stepped care approach to improving current “suboptimal” treatment of depression comorbid with a chronic physical health problem. [7] These clinical guidelines recommend that “for persistent subthreshold depressive symptoms or mild to moderate depression and a chronic physical health problem, ehealth interventions, in the form of computerised (or internet) cognitive behavioural therapy (iCBT) be used.” This recommendation was made on the basis of extrapolation from the iCBT literature in general: [7], however there is no evidence to directly support this recommendation in people with depression and physical health comorbidity. There are potential feasibility issues in that people with chronic physical conditions are typically older and thus less likely to be computer literate or comfortable using computers for health interventions. Only one randomised controlled trial [8], [9] has examined the efficacy of iCBT with adults over 50 years of age. It yielded promising results in group experiencing subclinical levels of depression, with an effect size comparable to group CBT treatment. No mention of comorbidity was made, although serious physical illness was an exclusion criterion.

We conducted a randomised controlled trial to evaluate the effectiveness of a freely accessible iCBT programme (*E-couch*) on depressive symptoms in people with at least mild to moderate depressive and elevated cardiovascular disease (CVD) and/or CVD risk factors. The secondary aim was to determine the effectiveness of the same intervention on adherence to treatment for cardiovascular disease and lifestyle advice. Tertiary objectives were to define impact upon anxiety symptoms, activity levels and disability.

## Methods

The protocol of the Cardiovascular Risk and *E-couch* Depression Outcome (CREDO) trial has been published elsewhere. [10] In summary, CREDO was an internet-based, double-blind, parallel group randomised controlled trial comparing the effectiveness of internet cognitive behavioural therapy (*E-couch*) with an online attention control (*HealthWatch*). The protocol for this trial and supporting CONSORT checklist are available as supporting information; see Protocol S1 and Checklist S1.

Participants were recruited through the *45 and Up Study,* a large-scale longitudinal population-based cohort study comprising over 260,000 men and women aged 45 years and over in New South Wales (NSW), Australia [11] recruited between 2006 and 2009. Comparative analysis of data from the *45 and Up Study* against the NSW Population Health Survey has demonstrated that, for a range of health and behavioural parameters the participants and associations found in the *45 and Up Study* are broadly representative of the general population. [12] From July 2010 to January 2011, 8000 participants who had met the following screening criteria according to their responses to the *45 and Up Study* questionnaire were invited to take part in CREDO:-.

1. Self-reported history of CVD, or risk factors for CVD, defined as any one of the following: receiving treatment for heart attack/angina, other heart disease, hypertension or high blood cholesterol in the past month; taking medications for heart disease, hypertension or high blood cholesterol in the past month; previous doctor’s diagnosis of heart disease, stroke or hypertension; previous doctor’s diagnosis of diabetes and report taking glucose lowering therapy in the past month; two or more of the following risk factors: current smoker, obese (body mass index >30), aged 65 years or more, family history of heart disease or stroke in two or more first degree relatives, all of which are well established risk factors for CVD. [13]
2. Psychological distress at recruitment to the health survey, defined with a high sensitivity as a Kessler-10 (K-10) [14] score of greater than or equal to 16. This screening score reflects distress six months to three years prior to trial recruitment.
3. Provided an email address established as previously valid through *45 and Up* Study checking processes.

Those screened as potentially eligible were invited by email to log on to a website where they were provided with study information, an opportunity to question the researchers, and online consent was obtained by indicating their agreement with two statements with yes/no response buttons. Once consented, they were directed to another webpage where they completed online baseline assessments and an automated cognitive assessment battery (CogState). [15] Participants were eligible for trial inclusion if they scored 8 or more on the Patient Health Questionnaire (PHQ-9) [16], [17] at this assessment, indicating persistent or recurrent symptoms. This criteria identifying at least mild-moderate depression, between the standard 5 (mild) and 10 (moderate) cutpoints [17] was chosen on the basis of pilot recruitment work to establish the lowest level at which recruitment would meet sample size requirements. Those who reported currently undergoing psychotherapy or expressed suicidal ideation on question 9 of the PHQ-9 at baseline were excluded from the trial. The latter went through a risk management protocol for further management. One week later participants were prompted by email to login to the trial site and confirm their continued willingness to participate and were randomised. Participants were blinded to which programme was the “active” intervention.

### Randomisation

Randomisation was undertaken using a customised, fully automated randomisation facility built into the trial website. Participants were randomised to receive either the intervention programme (*E-couch*) or the attention control programme (*HealthWatch*) and were stratified by depressive symptom severity using permuted block sizes of eight. Symptom severity was determined using PHQ-9 scores [16], [17], where scores ranging from 8 to 14 were classified as moderate, and scores ranging from 15 to 27 classified as major. Allocation was concealed from participants and researchers and assessments were all self report.

### Internet Delivered CBT Arm: E-couch

*E-couch* (http://www.ecouch.anu.edu.au/welcome) comprises 12 modules of psychoeducation (mental health literacy), cognitive behaviour therapy (CBT), and interpersonal psychotherapy (IPT) techniques that have evidence supporting their use in depression. The CBT and mental health literacy components of *E-couch* are extended versions of the *MoodGYM* [18] and *BluePages* [19] internet interventions, which have had effectiveness demonstrated in previous trials.[6], [18]–[23].

### Attention Control Arm: HealthWatch

*HealthWatch* is a 12-week online programme that delivers health information on topics including nutrition, stroke, physical activity, medicines in the home, blood pressure and cholesterol, and heart health to provide an attention matched control. In order to replicate the interactive component of the active treatment arm, participants also completed online questionnaires that probed their views about the importance of health factors, physical activities, hobbies, work habits and stress, medications, and alcohol use. Although providing valid health information, *HealthWatch* is a slightly extended version of the attention control that has previously been shown to have limited effects on depression [20].

### Programme Delivery

Modules in both interventions were made available sequentially weekly, with email updates. They have been reported to take between 30 and 60 minutes to complete in other studies. [18]–[23] Participants were not able to access the new module until they had completed the previous one. A link on the email provided support for technological problems. If a participant had not completed the current module within 4 days of its release, an automated reminder email was sent, followed by a phone call or text message three to four days after the reminder email if they had still not yet completed the new module. The phone calls did not provide any psychological intervention but did attempt to help the participant identify and overcome barriers to logging onto the site e.g. suggestions of how to incorporate the intervention into their schedule. If the participant had not completed three consecutive modules and not been contactable no further phone calls were made. This process was identical for both active intervention and attention control arms.

The investigators, analysts, trial manager and all participants were blind to treatment allocation for the duration of the trial. Any trial participant who reported suicidal ideation at any post baseline assessment episode underwent a risk management protocol by a blinded clinician for further management including a decision on whether to continue with the trial. The trial was registered with the Australian and New Zealand Clinical Trials Registry (ACTRN12610000085077) http://www.anzctr.org.au/ACTRN12610000085077.aspx.

### Outcome Measures

Assessments were conducted at baseline and post intervention (12 weeks), with additional data for the primary outcome obtained at 4 and 8 weeks.

#### Depressive symptoms

The primary prespecified outcome for the trial was severity of depressive symptoms from baseline to post intervention (12 weeks) measured using the PHQ-9; a nine-item assessment of depressive symptoms. This provides a summary score ranging from 0 to 27. The PHQ-9 has been found to be a reliable and valid measure, widely used in previous community studies of people with depression and is sensitive to change in clinical status. [16], [17].

#### Adherence

The prespecified secondary outcome of adherence to CVD treatment and lifestyle advice was measured using the “specific” component score of the Medical Outcomes Study (MOS) Measures of Patient Adherence Scale. This measure (score 0–100) is constructed from 15 questions assessing adherence to treatment and healthy behaviour recommendations e.g. exercise, diet and social support. It has been shown to be sensitive to changes in adherence and mood. [24] The more general (0–100) measure, comprising 4 questions about adherence to doctor’s advice, relies upon the individual having seen a doctor in the last four weeks and so was not applicable for many participants in this sample. The other prespecified secondary outcome was change in cognitive function at one year and will be reported when the twelve month outcomes are available.

Prespecified tertiary outcomes [10] were; anxiety measured using the Generalised Anxiety Disorder Scale (GAD-7), [25] activity (using the International Physical Activity Questionnaire (IPAQ), [26] specifically the average time spent walking per day and a composite measure of undertaking enough exercise to provide a health benefit (defined as at least 150 mins of activity over 5 or more occasions each week), and disability assessed by the WHODAS II (total score 0–100 and number of cutback days in last month). [27].

### Sample Size

The total study sample size of 470 was specified to detect a minimum difference of 0.3 standard deviations between groups in mean change in depressive symptom scores at the trial end point, with power of 90%, two-tailed α = 0.05, assuming a correlation of 0.5 between baseline and endpoint scores. Participation and eligibility rates and drop out from previous studies suggested that approximately 20% of those invited would consent to be assessed for trial participation, of whom 50% [18]–[20] would be ineligible and 20% would drop out over the study. Accordingly, 5650 were initially contacted. Due to a greater than expected number of emails not being current, a further 2350 were invited two months into the study recruitment. This increased the number of people attempted to be contacted to 8000, of whom 914 had a subsequent known failure through invalid details, leaving a potential sample of 7086.

### Analysis

Analyses were undertaken on an intention to treat (ITT) basis using mixed model repeated measures analysis of variance (MMRM) using the SPSS MIXED procedure. Within person variation was modelled using an unstructured covariance matrix with degrees of freedom estimated using the Satterwhaite approximation. A planned contrast evaluated differences from baseline to post-invention change between treatment arms. A post hoc analysis evaluated any outcome interaction with the presence or not of treated CVD. Where non-normality of residuals was found, appropriate transformations were applied. These yielded conclusions consistent with analyses of raw scores and thus only the results on familiar scales are presented. Other continuous outcomes were analysed using the same approach but, as there are only two time points for each test, the interaction was a direct test of the significance of differential change between conditions. Cohen’s d effect sizes for the difference between arms were calculated for each occasion of measurement using observed means and pooled post-intervention variance estimates, with confidence intervals calculated using the method described by Kelley. [28] Binary and ordered categorical outcomes were analysed using mixed effects logistic models analogous to the MMRM, including a random participant intercept implemented in gllamm in Stata 10.1.

Ethical approval was obtained from the University of Sydney Human Research Ethics Committee (Reference Number: 06-2009/11880) and from the Australian National University Human Research Ethics Committee (Reference number: 2010/085). The 45 and Up Study has primary ethical approval from the University of New South Wales Human Research Ethics Committee (HREC 05035).

## Results

Of 7086 invitations to potential participants, 1862 (26%) provided consent and proceeded to eligibility screening. Those consenting were more likely to be female (OR 1.11, 95% CI 1.05–1.19), speak English at home (OR 1.48, 95% CI 1.15–1.90) have a higher education (OR 1.67, 95% CI 1.46–1.92) and a prior doctor’s diagnosis of depression (OR 1.37, 95% CI 1.22–1.55): these variables were not associated with outcome amongst trial participants. [29] Only 696/1862 (37%) of those consenting met trial inclusion criteria, of whom 116/696 (17%) failed to complete baseline survey or return to website for randomisation and 18/696 (2.5%) withdrew consent before commencing leaving 562/696 (80.5%) who proceeded through to baseline assessment and randomisation (Figure S1).

Baseline characteristics were well matched between trial arms with no socio-demographic, or health differences (Table 1). There were no differences in baseline depression severity (PHQ-9 mean difference 0.2; 95%CI; −0.4–0.8) or the other outcome scores between arms (Tables 2 and 3). Participants had a mean age of 58 years, the majority were female (61.4%), married or partnered (72.6%), spoke English at home (94.5%) and had an education after basic schooling (72.7%). Over half (53.6%) had had a previous diagnosis of depression and 70% were already diagnosed and treated for existing cardiovascular disease. The remaining 30% had 2 or more other risk factors: obesity, being a current smoker, a strong family history of CVD or aged over 65. Obesity (BMI>30) was common (45.6%) but smoking uncommon (14.2%). The majority, 314/562 (56%) had another comorbid chronic physical health problem including cancer, thrombosis, osteoarthritis, Parkinson’s disease, asthma or thyroid disease.

**Table 1:** Baseline demographic and health characteristics of 562 participants randomised to either iCBT (*E-couch*) or attention control health information (*HealthWatch*).

	Active	Control
	(*E-couch*)	(*HealthWatch*)
	N = 280	N = 282
**Characteristics**		
**Continuous Measures**	**Mean (SD)**	**Mean (SD)**
Age in Years	57.5 (6.6)	58.4 (6.6)
**Categorical Measures**	**N (%)**	**N (%)**
Sex: Female	173 (61.8)	172 (61.0)
Speak English at Home: Yes	260 (92.9)	271 (96.1)
Marital Status: Partnered	203 (72.5)	205 (72.7)
Highest Qualification: Post-school	204 (72.9)	204 (72.6)
Private Health Insurance: Yes	173 (61.8)	196 (69.5)
Prior Diagnosis of Depression	150 (53.6)	151 (53.5)
Prior Diagnosis of Anxiety	91 (32.5)	88 (31.2)
Prior Diagnosis Cardiovascular Disease***	201 (71.8)	192 (68.1)
Treatment for any Cardiovascular Disease in Last Month##	194 (69.3)	199 (70.6)
Family History of CVD: Yes	188 (67.1)	191 (67.7)
Hazardous drinker		
Males (AUDIT-C score ≥5	47 (50.0)	52 (57.8)
Females (AUDIT-C score ≥4)	47 (36.7)	67 (46.9)
Current Smoker	41 (14.6)	39 (13.8)
Obese (BMI>30)	132 (48.7)	116 (42.5)
Exercise - Sufficient Time and Sessions$	145 (51.8)	149 (52.8)
One or more Comorbid Conditions#	157 (56.1)	157 (55.7)

* Scaled 0–100.

** Missing data for one participant who did not complete baseline assessment.

*** Prior Diagnosis of Cardiovascular Disease includes doctor diagnosis of any one of Heart Disease, Stroke or Hypertension.

$ At least 150 mins of activity over at least 5 sessions each week.

# Other comorbid conditions include: cancer (skin, prostate, breast or other cancer), blood clot (thrombosis), asthma, Parkinson’s disease, osteoarthritis, and/or thyroid problems.

## Treatment for any Cardiovascular Disease includes: any one of heart attack/angina, other heart disease, hypertension or high blood cholesterol.

**Table 2:** Observed depression, adherence, anxiety and disability measures for iCBT (*E-couch*) and active control (*HealthWatch*) at baseline and post intervention.

	Active (*E-couch*)		Control (*HealthWatch*)	
	N = 280 (at baseline)		N = 282 (at baseline)	
Outcome	Baseline	Post intervention	Baseline	Post intervention
	Mean (sd)			
Depression (PHQ-9)	12.0 (3.4)	8.4 (5.1)	11.8 (3.4)	9.2 (4.8)
Adherence (MOS Patient AdherenceScale – Specific)	24.3 (11.9)	26.8 (13.2)	24.3 (11.5)	25.0 (10.8)
Anxiety (GAD7)	8.9 (4.2)	6.5 (4.6)	8.7 (4.1)	7.3 (4.4)
Disability - WHODAS II total score	9.6 (5.3)	8.6 (5.8)	9.2 (5.1)	8.5 (5.1)

**Table 3:** Observed activity and disability (cutback days) measures for iCBT (*E-couch*) and active control (*HealthWatch*) at baseline and post intervention.

	Active (*E-couch*)		Control (*HealthWatch*)	
	N = 280 (at baseline)		N = 282 (at baseline)	
Outcome	Baseline	Post intervention	Baseline	Post intervention
	N (%)	N (%)	N (%)	N (%)
Activity*				
Sedentary	21 (7)	8(4)	21 (7)	18 (7)
Insufficient	114 (41)	60 (29)	112 (40)	89 (33)
Sufficient	145 (52)	136 (67)	149 (53)	165 (61)
Cutback days past month**				
0	91 (33)	78 (38)	94 (33)	99 (36)
1/7	85 (30)	63 (31)	87 (31)	84 (31)
8+	103 (37)	62 (31)	101 (36)	89 (33)
Total walking time				
0–14 min per day	138 (49)	78 (38)	144 (51)	116 (43)
15–29 min per day	63 (23)	47 (23)	56 (20)	64 (23)
30+ min per day	79 (28)	79 (39)	82 (29)	92 (34)

* - “sedentary” defined as reporting no physical activity per week.

- “sufficient activity” defined as at least 150 mins of activity over at least 5 sessions each week.

** - cutback days defined as number of days in last month where respondent reported “cutting back or reducing their usual activities or work as a result of a health condition”.

Of trial participants, 260/280 (93%) assigned to iCBT and 278/282 (99%) assigned to the control arm provided at least one post baseline measure. All participants contributed to the analysis in the ITT modelling approach. Complete post intervention depression scores were available for more of those in the control arm (97%) than those assigned iCBT 76%), (Odds ratio 9.36, 95% CI: 6.36–13.75). Baseline depression severity (PHQ-9 score) was not associated with study dropout overall (Odds ratio = 0.97, p = 0.43, 95% CI: 0.90–1.05) but in the iCBT arm a linear association of baseline depression severity and dropout approached significance (Odds ratio = 0.92, p = 0.05, 95% CI: 0.84–1.001). The direction of the association implies that participants with higher levels of depression at baseline were less likely to withdraw from the trial. There was no depression-attrition association in the control arm. Attrition was not associated with any demographic factor or outcome except that it was more common amongst the small group (n = 31 (5.5%)) who did not speak English as their first language at home.

The observed results for all outcomes at baseline and post intervention are presented in Tables 2 and 3 and the estimated means and contrasts from the MMRM approach in Table 4.

**Table 4:** Baseline to post-intervention change under iCBT (*E-couch*) and control (*HealthWatch*) on depression, adherence, anxiety, disability and activity measures.

	Baseline–post intervention change		Interaction	
Outcome	Active(*E-couch*)	Control (*Health­Watch*)	Difference	Test
Depression (PHQ-9)	3.66 (3.05–4.27)	2.60 (2.05–3.16)	1.06 (0.23–1.89)	P = .01
Adherence (MOS Patient Adherence Scale – Specific)	2.72 (1.35–4.09)	0.56 (−0.65–1.77)	2.16(0.33–3.99	P = .02
Anxiety (GAD7)	2.44 (1.87–3.02)	1.48 (0.97–1.99)	0.96 (0.19–1.73)	P = .01
Disability - WHODAS II total score	1.08(0.55–1.61)	0.68 (0.22–1.44)	0.40(−0.30–1.10)	P = .26

All contrasts are scaled so that a positive value indicates improvement.

Contrast value (95% Confidence Interval).

### Primary Outcome - Depression Symptom Severity (Tables 2 and 4, Figure S2)

Participants in both arms showed symptomatic improvement by, on average (observed means) 3.66 (95% CI: 3.05–4.27) points with iCBT and 2.60 points (95%CI 2.05–3.16) in the control group, with a significantly greater decline in the PHQ-9 for iCBT compared to control (1.06; 95%CI: 0.23–1.89; time by arm interaction p = .012). The effect was seen both those being treated for CVD and those just with a higher risk status :the time by condition by CVD treatment interaction was not significant, indicating no reliable differential effects (F test for the interaction is F(3,505.8) = 0.76, p = 0.5078). This difference was slightly less than that hypothesised: 0.22 standard deviations of the mean observed change score.

### Secondary Outcome - Adherence to Medical and Lifestyle Intervention (Tables 2 and 4)

Specific adherence scores were available for all participants at baseline and at follow-up for 204/280 (73%) participants in the iCBT arm and 272/282 (96%) participants assigned to the control group. Assignment to iCBT resulted in adherence scores higher by 2.16 points (95% CI: 0.33–3.99). Only 2 participants reported no medical or lifestyle intervention behaviours at all.

### Tertiary Outcomes (Tables 2–4)

The iCBT resulted in greater anxiety score (GAD-7 [23]) improvement by 2.44 (95%CI: 1.87–3.02) points with iCBT compared to 1.48 (95%CI:0.97–1.99) in the control group with a difference in means of 0.96 (95% CI: 0.19–1.73) points (time by arm interaction p = 0.014). There was a greater proportion engaging in activity sufficient to confer a health benefit post intervention (150 mins over 5 occasions/week); 67% in iCBT vs. 61% in the control group (Odds Ratio 1.91, 95%CI: 1.01–3.61), but no differential improvement in average walking time per day: time by arm interaction (Odds ratio = 1.46, 95% CI: 0.81–2.62), (table 3). Although disability improved slightly in both groups (iCBT 1.08 (95% CI: 0.55–1.61), Control: 0.68 (95% CI: 0.22–1.14) there was no differential advantage for iCBT: (mean difference 0.40 (95% CI: −0.30–1.10), time by arm interaction p = 0.262) or when assessed as cut back days (Odds ratio = 0.82, 95% CI: 0.48–1.41), (table 3).

### Adverse Outcomes

After enrolment 44 participants reported a score of 2 or more on item 9 of the PHQ-9 indicating a risk of deliberate self harm: 21 in the iCBT arm and 23 in the control arm All of these were contacted by clinicians, masked to treatment allocation, as part of the risk management protocol. None were deemed acute clinical risks and none were removed from the study.

## Discussion

This trial demonstrates that an iCBT program (*E-couch*) can improve both mood and self reported adherence in people with mild to moderate depression and physical health problems, and more so than internet delivered health and lifestyle advice. The majority of the participants had existing, treated cardiovascular disease, and more than half another chronic physical problem, with no differential treatment effects between those with or without treated CVD. While the effects are modest in size, the intervention has the potential to be applied as a low intensity psychosocial intervention to large numbers of people. In the UK for instance current NICE guidelines for people with comorbid depression and physical health problems [7] recommend iCBT for this purpose by extrapolation from trials in younger people or people in whom health problems are not a key feature. Although by no means a panacea, *E-couch* is freely available and this trial provides the first evidence to confirm the effectiveness of such an approach, supporting these guidelines.

### Strengths and Weaknesses of the Study

The trial recruited participants from a large health survey who, despite the majority having physical disease, may have health behaviours systematically different to that in the general population. [30], although in this trial no self-sampling bias with respect to outcome could be demonstrated. [29]The inclusion definitions were based entirely upon self report which could have led to some misclassification but some aspects of the 45 and Up study self report data have been validated [31]. However there was no differential effect on those being treated for CVD (where self report may be more reliable) and those with only a higher risk profile which makes our results more generalisable. Any end point completion rate was moderately differential for the primary outcome (93% for iCBT and 99% for control) and a lower post test completion rate in the intervention arm (214/280 (76%) vs. 273/282 (97%)) (χ^2^ = 50.46, df = 1, p<.001) by three months, with a trend for those who dropped out in the iCBT arm to have had milder depressive symptoms at baseline (Odds ratio = 0.92, 95% CI: 0.84–1.001). It thus seems unlikely that the improved depression outcome observed for iCBT at follow-up can be ascribed to the differential loss of patients with milder disease at baseline and the true effect of the intervention may be greater than that recorded here. We cannot discount that both interventions hampered improvement. However given that the precursor to E-couch (MoodGym) has been shown to be more effective than a waiting list control [22] this seems unlikely.

This effect of a higher drop out in the intervention arm of ehealth studies seems common: A review of adherence and persistence to internet treatments for depression [28] found that most studies report lower rates of completion in the experimental intervention group relative to the control, with suggestions that the greater demands of the active programme interaction are less tolerable. Qualitative studies [32] attempting to understand this process have highlighted both the obvious barriers e.g. extra time requirements often in the active arm and also the need to enhance the therapeutic relationship perception and provide feedback about progress. However overall, this trial achieved better retention in terms of assessment completion than have almost all previous trials of iCBT [33] even though the blinded research assistants provided only technical assistance with no clinical support, as would occur in large scale implementation. There were however several reminders and some personal contact which could have increased the effect of the intervention [4]. However any such effect would also have been exerted on the attention control arm where the same level of reminders and phone contact was implemented and thus be unlikely to systematically bias the results. We assume that there is some minimal level of adherence to the programme required to achieve the desired outcomes although this level has not yet been established. [34] The traditional clinical trial and evidence-based medicine paradigm stipulating that high dropout rates make trials less believable does not necessarily apply to such interventions where there is not necessarily a dose response relationship. [35] A more nuanced understanding of the poorer persistence in the more demanding active arm, and whether this even needs addressing, is required.

Many previous studies have shown an effect of iCBT interventions, including the precursor to this programme (MoodGYM) and Beating the Blues, [6], [17]–[23] but only one has done so in people aged over 50 [8], [9] and none have targeted people with comorbid physical health problems. The effect size for the active vs. control arm in improving depression symptom severity in this trial was only small, at 0.15. This does however compare reasonably with the small to moderate effect sizes of psychological interventions for depression in people with cardiovascular disease recruited from hospital settings (standardised mean difference (SMD) 0.21, 95% CI: 0.08–0.35) observed in the latest systematic review. [36] In patients with diagnosed major depressive disorder (MDD) a decrease of five points on the PHQ9 has been suggested as clinically significant [17]. The rates of such a “clinically significant” change were greater with iCBT, but not statistically significantly (relative risk of change by post test (1.21 (95% CI: 0.94–1.55). However only 153/562 (27%) met MDD diagnostic criteria, the rest having milder forms of depressive symptoms which may introduce floor effects in the use of such categories, whereby smaller changes may be meaningful e.g. a reduction of a score from 9 (mild) to 5 is only a change of four points yet results in someone effectively having no meaningful symptom load.

Observational studies have suggested that improvement in post-MI depression is associated with greater adherence to aspirin [37] and other behavioural interventions, [24] the latter study using the same self report measure as here. This study however provides the first trial evidence that an intervention that reduces depression may have additional self reported adherence benefits. There is a potential limitation in relying upon self report which may reflect a reporting bias. Future studies would benefit from objective adherence measures. Of note, those in the control condition reported no change in adherence to health and lifestyle advice such as exercising regularly, cutting down on salt, and following medication recommendations despite the control arm focussing specifically on providing information on the health impacts of such behaviours. This suggests that attempts to enhance the engagement with lifestyle and medical interventions cannot just rely upon information provision. [32], [38]The cognitive behavioural techniques and processes, such as behavioural review and goal setting, embedded in iCBT, seem to provide a more effective approach.

### Conclusion

In summary, this trial provides the first evidence that a freely available internet delivered CBT program (*E-Couch*), with minimal personal support, is effective as a low intensity psychological intervention for people with the levels of depressive symptoms seen commonly in primary care and comorbid CVD risks and disease. Compared to an attention control providing health and lifestyle information it improved both psychological symptoms and, importantly if replicable, self reported adherence to medical and lifestyle interventions and some aspects of activity. This is the first direct empirical support for the guideline recommendations for such interventions e.g. current UK NICE guidelines. [7] These findings require replication in different settings, particularly focussing on participants with other comorbidities. We do not know if the reported improvement in adherence actually reflects objective adherence and will translate into better CVD outcomes. Determining the most cost effective technical and personal support processes to enhance engagement [28], [30] and its place in current public health or clinical care models should be the next avenue of investigation.

## Supporting Information

Figure S1 **Consort Flow Diagram.**
(TIF)

Figure S2 **Observed Depression (Mean PHQ 9 Total) Scores by treatment arm at each occasion of measurement.** (Error bars are ±1 standard error.)
(TIFF)

Checklist S1 **CONSORT Checklist.** CREDO Consort 2011 Checklist.
(DOCX)

Protocol S1 **Trial Protocol.** CREDO protocol.
(PDF)
