# Supplementary figures and images for: Internet-Delivered Cognitive Behavioural Therapy for Adults with Mild to Moderate Depression and High Cardiovascular Disease Risks: A Randomised Attention-Controlled Trial
Source: PLoS One. 2013 Mar 26;8(3):e59139. doi: 10.1371/journal.pone.0059139 (PMC3608590; doi:10.1371/journal.pone.0059139)

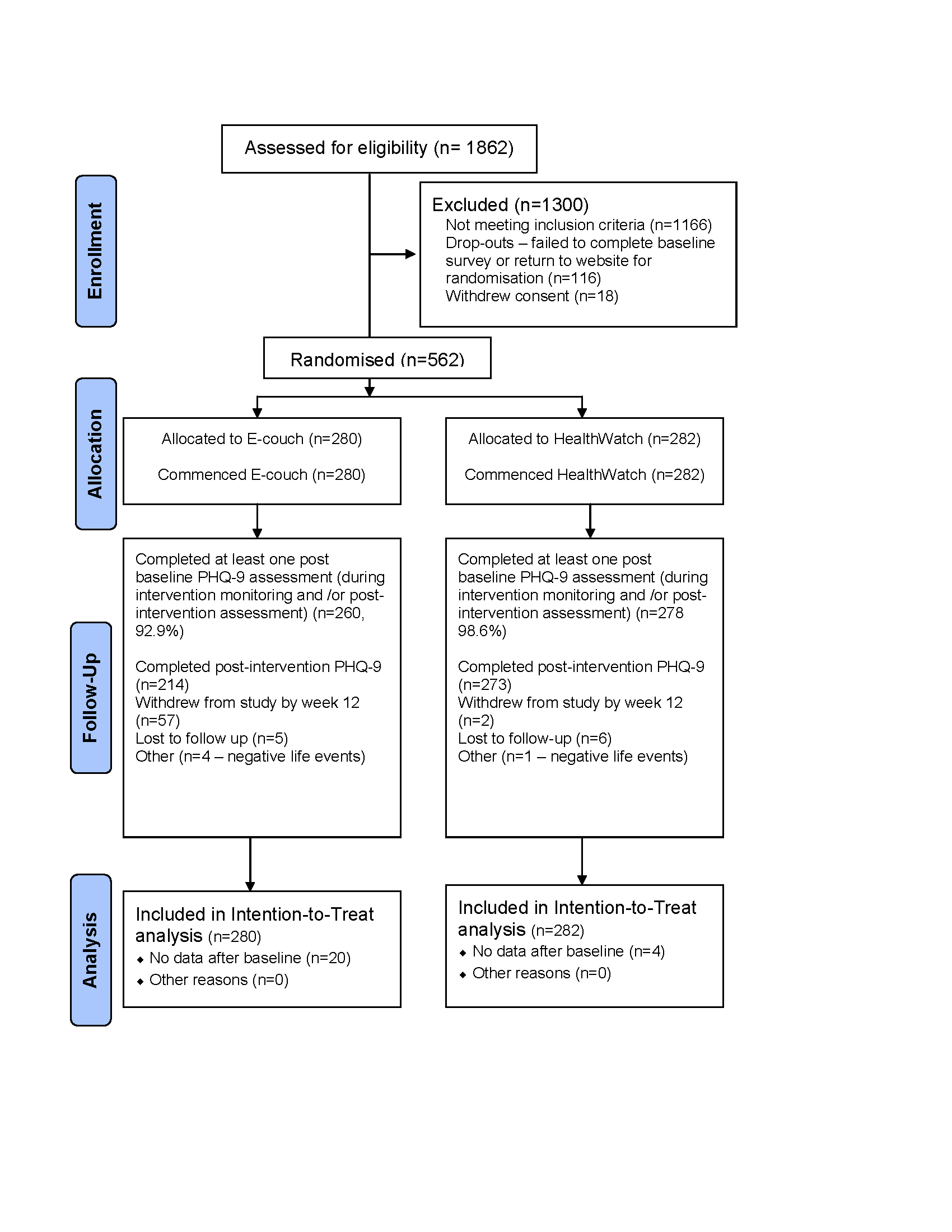

Supplement: Figure S1 — Consort Flow Diagram. (TIF) [file pone.0059139.s001.tif]

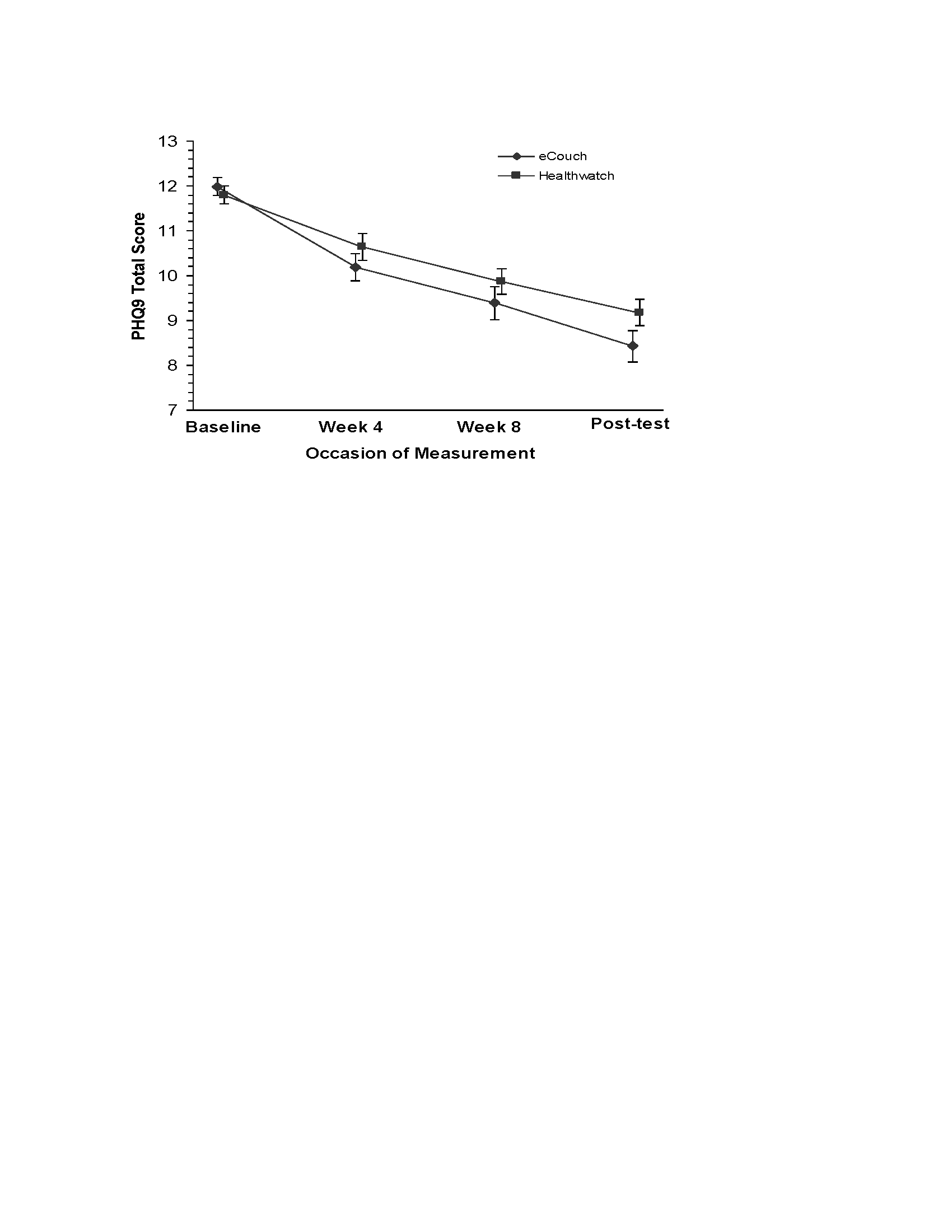

Supplement: Figure S2 — Observed Depression (Mean PHQ 9 Total) Scores by treatment arm at each occasion of measurement. (Error bars are ±1 standard error.) (TIFF) [file pone.0059139.s002.tiff]
