# Supplementary material for: Internet-Delivered Cognitive Behavioural Therapy for Adults with Mild to Moderate Depression and High Cardiovascular Disease Risks: A Randomised Attention-Controlled Trial
Source: PLoS One. 2013 Mar 26;8(3):e59139. doi: 10.1371/journal.pone.0059139 (PMC3608590; doi:10.1371/journal.pone.0059139)
Supplement: Protocol S1 — Trial Protocol. CREDO protocol. (PDF) [file pone.0059139.s004.pdf]

Cockayne et al. BMC Psychiatry 2011, 11:10
http://www.biomedcentral.com/1471-244X/11/10

BMC Psychiatry

STUDY PROTOCOL Open Access

# Internet-based treatment for older adults with depression and co-morbid cardiovascular disease: protocol for a randomised, double-blind, placebo controlled trial

Nicole L Cockayne[1], Nick Glozier[1,2*], Sharon L Naismith[1], Helen Christensen[3], Bruce Neal[4], Ian B Hickie[1]

## Abstract

**Background:** Depression, cardiovascular disease (CVD) risk factors and cognitive impairment are important causes of disability and poor health outcomes. In combination they lead to an even worse prognosis. Internet or web-based interventions have been shown to deliver efficacious psychological intervention programs for depression on a large scale, yet no published studies have evaluated their impact among patients with co-existing physical conditions. The aims of this randomised controlled trial are to determine the effects of an evidence-based internet intervention program for depression on depressive mood symptoms, cognitive function and treatment adherence in patients at risk of CVD.

**Methods/Design:** This study is an internet-based, double-blind, parallel group randomised controlled trial. The trial will compare the effectiveness of online cognitive behavioural therapy with an online attention control placebo. The trial will consist of a 12-week intervention phase with a 40-week follow-up. It will be conducted in urban and rural New South Wales, Australia and will recruit a community-based sample of adults aged 45 to 75 years. Recruitment, intervention, cognitive testing and follow-up data collection will all be internet-based and automated. The primary outcome is a change in severity of depressive symptoms from baseline to three-months. Secondary outcomes are changes in cognitive function and adherence to treatment for CVD from baseline to three, six and 12-months.

**Discussion:** Prior studies of depression amongst patients with CVD have targeted those with previous vascular events and major depression. The potential for intervening earlier in these disease states appears to have significant potential and has yet to be tested. Scalable psychological programs using web-based interventions could deliver care to large numbers in a cost effective way if efficacy were proved. This study will determine the effects of a web-based intervention on depressive symptoms and adherence to treatment among patients at risk of CVD. In addition it will also precisely and reliably define the effects of the intervention upon aspects of cognitive function that are likely to be affected early in at risk individuals, using sensitive and responsive measures.

**Trial registration:** Australian New Zealand Clinical Trials Registry (ANZCTR): ACTRN12610000085077

## Background

Ischaemic heart disease and major depression are the two leading causes of disease burden as measured using disability-adjusted life years in OECD countries, contributing 9.0% and 6.8% of the total burden respectively. The associated conditions of stroke, diabetes and alcohol abuse are also among the top ten causes of disease burden [1]. Dementia contributes a further 2.9% and is increasing in the ageing Australian population.

The World Mental Health study demonstrated that the individual health decrement (disability) arising from depression-physical disorder co-morbidity produces

* Correspondence: nick.glozier@sydney.edu.au
[1]Brain & Mind Research Institute, The University of Sydney, 100 Mallet Street, Camperdown NSW 2050, Australia
Full list of author information is available at the end of the article

© 2011 Cockayne et al; licensee BioMed Central Ltd. This is an Open Access article distributed under the terms of the Creative Commons Attribution License (http://creativecommons.org/licenses/by/2.0), which permits unrestricted use, distribution, and reproduction in any medium, provided the original work is properly cited.

greater disability than would be expected from purely additive effects [2]. Furthermore, treatments are typically targeted only at those with major depressive disorder although the public health impact of lesser symptom severity is highly significant [3,4]. The large numbers with sub-clinical or 'subsyndromal' disease contribute a greater disease burden than the few with severe illness [5]. Providing acceptable, non-toxic interventions on a large scale for those with less severe depressive symptoms in the community is therefore a key public health goal.

The relationships between depression and cardiovascular disease (CVD) are complex with bidirectional pathways [6]. Depression has been shown to have a relatively strong association with the development of fatal coronary heart disease as well as myocardial infarction. This finding has been demonstrated by a number of longitudinal studies published over the last 40 years, is particularly true in men [7] and is apparent across the range of depression symptom severity [7].

Although depression is consistently associated in observational studies with poor outcome in people with both established CVD and with risk factors for CVD [7], all major randomised controlled trials (RCTs) have evaluated the effect of treating depression only in those with an *established event* such as myocardial infarction. These trials aiming to treat depression and thus reduce CVD have produced mixed results. Despite demonstrating reasonable evidence for an antidepressant response in a number of studies [8-10], no benefits were found in prevention of recurrent events or cardiac death. This was also true of the most recently reported trial (CREATE; [11]). There remains, however, the possibility that intervention in people with less severe disease (i.e., either less severe depressive symptoms or cardiovascular risk factors) might be of benefit [12]. For example, people at high risk for CVD (e.g. raised cholesterol, smokers) but without established disease may have suboptimal medical management, in addition to psychosocial factors, which may play a greater role in factors such as adherence. We propose that new interventions need to target these 'at risk' individuals.

Poor adherence to medications is an issue that is often linked with depression in conceptual papers but rarely addressed in clinical studies. Adherence describes a person's implementation of a health related behavioural prescription, such as correctly taking a medication, or exercising according to an agreed plan. Depression is one the most consistent determinants of poor adherence to physical treatments [13] and medication [14]. Poor adherence is often postulated to mediate the effects of depression on cardiovascular outcomes. To date, however, there has been no RCT evaluating whether an intervention designed to improve depression in those with co-morbid CVD also improves adherence to preventative therapies.

In addition to the range of poor physical health outcomes, depression is also associated with impaired cognition. Typically, dysfunction is evident in those regions mediated by fronto-subcortical brain circuitry, with impairments being most pronounced in processing speed, executive functioning and memory [15]. These impairments are predictive of disability and poor quality of life [16] and often persist despite symptom resolution [17]. While the precise mechanisms are unknown, cognitive impairment in older people with depressive symptoms may be due to a combination of underlying cerebrovascular disease, as well as the direct neurotoxic effects of depression itself. This research underscores the need to deliver targeted interventions for these modifiable risk factors as early as mid-life [18]. To-date, no known trials have evaluated the effect of treating depressive symptoms upon the subtle early cognitive impairments observed in this 'at risk' group.

Taken together, the literature on depression and CVD would suggest that there exists a group of individuals with both higher cardiovascular risk and depressive symptoms whose coexistence can initiate a negative spiral towards worse CVD and mental health outcomes. Taken in turn, this is likely to have deleterious effects on cognitive functioning, and levels of disability. Given the rapidly, ageing population, there is a need to address these issues on a large-scale at primary and secondary levels of prevention. Internet or web-based interventions (e-health) may be ideally suited for this purpose since they have been shown to deliver efficacious psychological intervention programs for depression [19] on a large scale in a cost effective manner. Moreover, there is evidence that internet interventions are preferentially sought for their anonymity, their capacity to be used privately at home and for their lack of face-to-face contact. As such they may increase participation among individuals who might not otherwise seek care [20]. Middle-aged men, a key target group for early cognitive decline, are particularly hard to engage in standard health care. Internet interventions - if automated - are able to deliver interventions with fidelity, giving them an advantage over other types of programs. In younger samples, data suggests that internet interventions are effective for a range of mental health symptoms including depression, post traumatic stress disorder, and eating disorders [21] and some studies have shown sustainable benefits [22]. To-date, there have been no published evaluations of web-based treatments for mood and co-morbid physical disorders in older people, although trials in younger samples appear to be underway [23].

The primary aim of the Cardiovascular Risk, E-couch Depression Outcome (CREDO) research trial is to determine the efficacy of an internet intervention program for depression (*e-couch*) on depressive symptoms in

people being treated for, or at risk of developing CVD. The secondary aims are to determine the immediate, six and 12-month efficacy of the same intervention on cognitive function and adherence to treatment for CVD.

## Methods/Design

### Study Design

This study is a randomised, double-blind, controlled trial of parallel design. Participants will be randomly allocated to one of two groups: *e-couch*, an online program that primarily provides cognitive behavioural therapy (CBT) yet also incorporates modules on interpersonal psychotherapy (IPT), relaxation and physical activity; and *HealthWatch*, an online active control program. The trial consists of a 12-week intervention treatment phase with a 40-week follow-up phase. The total trial period will be 12-months. As shown in Figure 1, measurements will be undertaken at four time-points in each group: at baseline, directly after completing the 12-week internet program, and at six and 12-month follow-up.

### Participants and Setting

#### *Sample*

The target population for the trial are older adults, both male and female aged 45 to 75 years, with self-reported CVD history or significant risk factors, and evidence of depressive symptoms.

Participants will be recruited through the *45 and Up Study*, a large-scale longitudinal population-based cohort study comprising over 260,000 men and women aged 45 years and over in New South Wales (NSW), Australia [24]. Participants in the *45 and Up Study* were randomly selected from the database that is used to administer the national universal health insurance scheme (Medicare Australia), which has almost complete coverage of the Australian population. Participants entered the study by completing a baseline postal questionnaire and providing written consent to have their health followed over time. The study questionnaire is available at http://www.45andUp.org.au The overall response rate to the baseline questionnaire is 18%. Comparative analysis between the *45 and Up Study* and the NSW Population Health Survey demonstrates that for a range of risk factors the estimates of relative risk observed in the *45 and Up Study* are generalisable to the wider population [25].

#### *Recruitment*

Potential participants will be randomly selected from the *45 and Up Study* database using an algorithm to identify those that meet the eligibility criteria and have provided a valid email address. The individuals identified will be contacted via email, given brief information about the trial and provided with a Trial ID and a link to the trial website. Interested participants will log into the website using their Trial ID where they will be provided with more detailed information and undertake an online consent, eligibility screening and baseline assessment procedure. Recruitment will be conducted in waves of approximately 1,000 until the desired sample size is reached.

#### *Eligibility Criteria*

Inclusion criteria: To be eligible to be screened for participation in the trial, individuals must have an email address and have satisfied the following two criteria in the *45 and Up Study* baseline questionnaire:

1. Self-reported history of CVD, or risk factors for CVD, defined as any one of the following:

   - Receiving treatment for heart attack/angina, other heart disease, hypertension or high blood cholesterol in the past month;
   - Taking the following medications listed in the *45 and Up Study* questionnaire for heart disease, hypertension or high blood cholesterol in the past month: Lipitor, Pravachol, aspirin for the heart, Avapro, Karvea, Coversyl, Coversyl Plus, Cardizem, Vasocardol, Norvasc, Tritace, Noten, Tenormin, atenolol, warfarin, Coumadin, Lasix, frusemide, and Micardis;
   - Previous doctor's diagnosis of heart disease, stroke or hypertension;
   - Previous doctor's diagnosis of diabetes and report taking glucose lowering therapy in the past month;
   - Two or more of the following risk factors: current smoker, obese, aged 65 years or more, family history of heart disease or stroke in two or more first degree relatives.

2. Self-reported psychological distress defined as a Kessler-10 (K-10) score of greater than or equal to 16.

In addition, at the time of eligibility screening, individuals must have a score of 8 or above on the Patient Health Questionnaire (PHQ-9), thus demonstrating the presence of clinically significant depressive symptoms at the point of entry to the trial.

Exclusion criteria: Those excluded will be individuals with either no depressive symptoms, or those that express suicidal ideation as determined from the PHQ-9. Also, those individuals that report they are currently receiving any form of counselling, e.g., with a counsellor, general practitioner, psychiatrist or psychologist will be excluded. Refer to Table 1.

### Procedure

The Participant Information Statement and Consent Form will be available on the trial website and available for download. The Participant Information Statement provides a detailed description of the trial, the procedures and time involved in participating, information on the randomisation process each participant will undergo

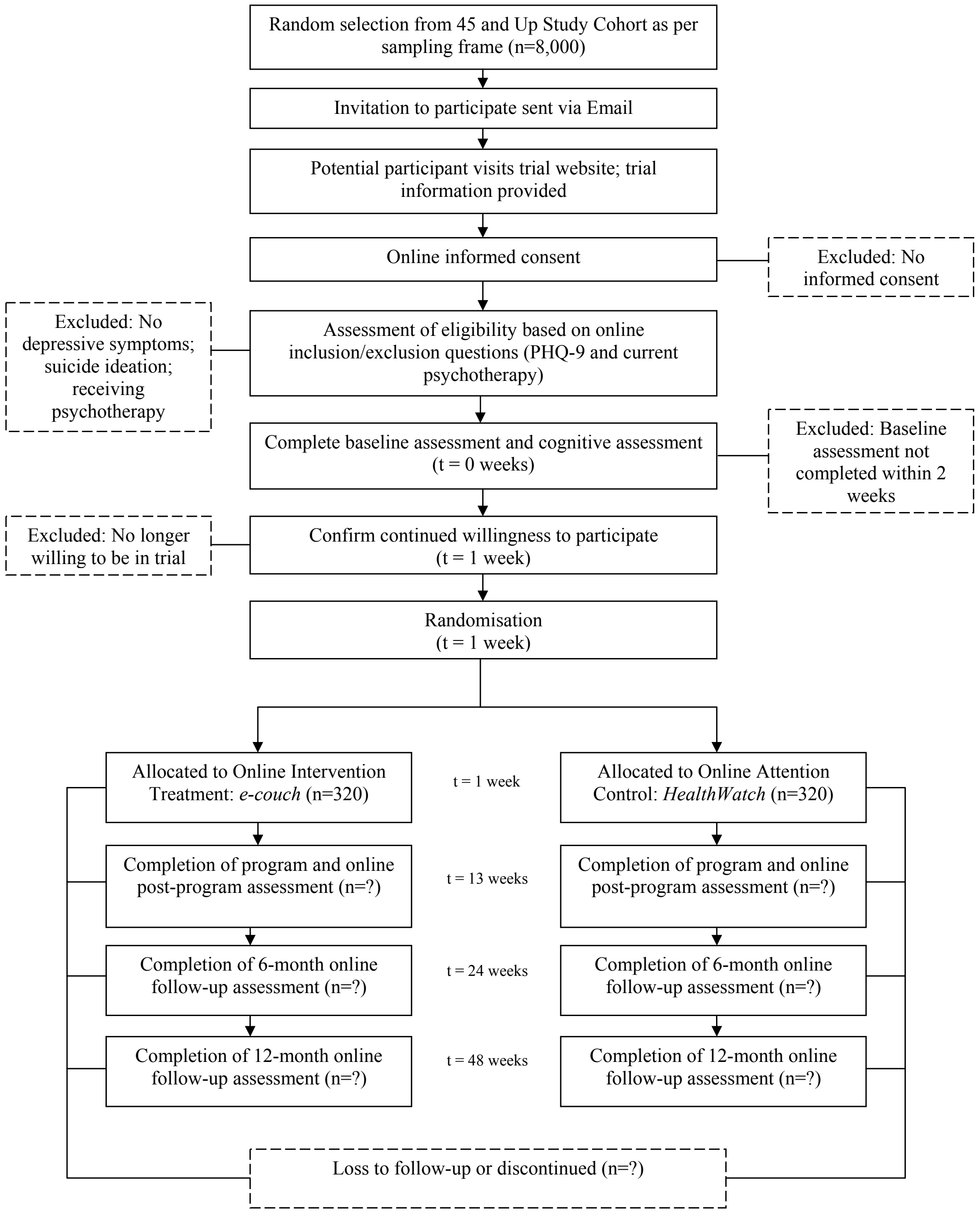


**Figure 1 Flow of Participants**.

to either one of two interactive internet programs, confidentiality information and contact information for the trial team should potential participants want further information.

In providing consent, within the online form participants will be required to: enter their full name; indicate their agreement with two statements by checking a yes/no response button and then; click on submit. The date consent is submitted will be recorded.

Once online consent is obtained the potential participant will complete the PHQ-9 and questions relating to any form of counselling currently being received. Participants deemed to be eligible and will be asked to complete the baseline data collection which comprises

**Table 1 Inclusion/Exclusion Criteria**

| | |
|---|---|
| **Inclusion Criteria** | |
| Self-reported history of any **one** of the following CVD or risk factors in the 45 and Up Study baseline dataset | Report of treatment in the last month for:<br>• Heart attack/angina<br>• Other heart disease<br>• High blood pressure<br>• High blood cholesterol |
| | Report of taking any of the following in past four weeks:<br>• Lipitor<br>• Pravachol<br>• Aspirin for the heart<br>• Avapro/Karvea<br>• Coversyl/Coversyl Plus<br>• Cardizem/Vasocardol<br>• Norvasc<br>• Tritace<br>• Noten<br>• Tenormin/atenolol<br>• Warfarin/Coumadin<br>• Lasix/frusemide<br>• Micardis |
| | Previous doctor's diagnosis of heart disease or stroke or high blood pressure |
| | Previous diagnosis of diabetes and taking at least one prescription of glucose lowering therapy in the last four weeks |
| | Two or more of the following risk factors:<br>• Regular smoker now<br>• Obese (Body Mass Index ≥ 30)<br>• Over 65 years<br>• Family history of heart disease or stroke in two or more first degree relatives |
| Self-reported psychological distress in the 45 and Up Study baseline dataset | A Kessler-10 (K-10) score of greater than or equal to 16. |
| Depressive symptoms indicated at pre-trial screening | A score of eight or above on the 27 point Patient Health Questionnaire (PHQ-9) |
| **Exclusion Criteria** | |
| No depressive symptoms at pre-trial screening | A score less than or equal to seven on the 27 point scale of the PHQ-9 |
| Suicide ideation at pre-trial screening | A score of either '2' or '3' on the 0-3 likert scale at question nine of the PHQ-9 |
| Currently receiving psychotherapy at pre-trial screening | Indication that currently receiving any form of counselling e.g. with a counsellor, GP, psychiatrist or psychologist |

a survey and cognitive test battery; *CogState*. Participants will be encouraged to complete both the baseline survey and cognitive tests within one week. Within one day of completing their baseline assessment, participants will receive an automated email thanking them for their involvement and confirming the next steps of the trial. Ongoing participation in the trial will be measured from this time-point. Refer to Figure 1.

Eligible participants that complete the baseline assessment will be prompted with an automated email one week later asking them to return to the trial website to commence their internet program. Participants will be required to login and confirm their continued willingness to participate in the trial. The process of confirming their willingness to participate will automatically trigger the randomisation facility built into the internet program. Refer to Figure 1.

#### *Randomisation*

Randomisation will be undertaken using a customised and fully automated randomisation facility built into the trial website. Participants will be randomised to receive either the intervention program (*e-couch*) or the active control program (*HealthWatch*). Randomisation will be stratified by depressive symptom severity using specified block sizes of eight. Symptom severity will be determined using PHQ-9 scores, where scores ranging from 8 to 14 are classified as moderate depressive symptoms, and scores ranging from 15 to 27 are classified as major depressive symptoms. The computerised procedure allows for full replication.

### Interventions

#### *Active Treatment Arm: e-couch*

*E-couch* is an automated software program that offers 12 modules addressing mental health literacy (information about the nature, risk factors and effective treatments for depression), CBT, IPT, relaxation techniques and exercise programs targeting depression. The CBT and mental health literacy components of *e-couch* are extensions of the *MoodGYM* [26] and *BluePages* [27] internet interventions, which have efficacy demonstrated in previous trials [28]. Both of these programs have been found to reduce depression symptoms in community samples relative to placebo conditions [19,29].

#### *Active Control: HealthWatch*

*HealthWatch* is a 12-week program in which participants read information about environmental health, nutrition, stroke, physical activity, medicines in the home, temperature extremes, oral health, blood pressure and cholesterol, heart health, bacteria and food-borne illnesses, calcium and back pain. In order to replicate the interactive component of the active treatment arm, participants also complete online questionnaires that probe health factors, physical and artistic activities, education and hobbies, social, financial, and family roles, work habits and stress, medications, pain and nutrition, and alcohol use.

#### *Intervention Procedure*

Modules are made available sequentially weekly and take between 30 and 60 minutes to complete. An automated

email at the commencement of each week will notify participants that their weekly module is available for completion. A link on the email provides support for technological problems. In each program, one new website module will open each week regardless of whether a participant has completed the previous one. For example, at Week three, module three will be released. If a participant has only completed module one, but logs on in Week three they will be directed to complete Week two first. However, they will not have to wait a week between each of these modules since Week three will have already been released. Each module will remain open after it has been completed so that participants may go back and revise any information.

If a participant has not logged in to the website and completed the current module within four days of its release, an automated reminder email will be sent. A phone call will then be made three to four days after the reminder email has been sent if they have still not yet completed the new module.

### Participant Monitoring and Follow-up Assessments

Participants will be monitored as the trial progresses with questions relating to mood embedded within the respective internet programs in Week four and Week eight. Module completion will be monitored each week, with automated reminder emails and follow-up phone calls undertaken as outlined above.

Upon completion of the trial treatment program, participants will be prompted by automated email to return to the trial website to complete the post-program assessment, comprising a survey and cognitive testing. Phone calls will be made to participants that do not respond to the automated email notification to encourage completion. Further follow-up assessments will be completed at six and 12-months respectively following date of randomisation. Refer to Figure 1.

### Data Collection

Reasons for exclusion and withdrawal or loss to follow-up will be recorded. Table 2 provides an overview of the timeframe for assessment and the measures that are used. Data collection for the trial will be done online with questions embedded in the trial website and in the respective intervention programs or collected via the *CogState* (cognitive testing) program. At six and 12-month follow-up, where a participant no longer has access to the internet, the survey component of the assessment will be conducted via phone by blinded interviewers.

### Outcome Assessment

#### *Primary Outcome - Depressive Symptoms*

The primary outcome for the trial is a change in severity of depressive symptoms from baseline to three, six and 12-months. The primary outcome will be measured using the PHQ-9; a nine-item assessment of depressive symptoms, which provides a summary score ranging from 0 to 27. The PHQ-9 is a reliable and valid measure of depressive symptoms, which has been widely used in previous studies of people with depressive symptoms and is sensitive to change [30,31]. The PHQ-9 will be embedded into the trial website and completed online at baseline, post-program (three-months) and six and 12-month follow-up. Mean changes in continuous depressive symptom scores from baseline will be compared between the two randomised groups at each time-point.

#### *Secondary Outcomes - Cognition and Adherence*

Cognition: Cognitive function will be measured using *CogState*, a computerised test battery with well established validity and sensitivity to detect subtle cognitive change in community cohorts [32-35]. For this trial, an internet deliverable version will be utilised. The test battery was chosen for brevity as well as capacity to probe psychomotor speed, memory and executive functioning. Specifically the battery will take a maximum of 12-minutes to complete and includes:

a) Psychomotor function/speed of processing: This task requires participants to respond when a card presented on-screen turns from face-down to face-up (duration, two minutes) (outcome measure = speed of performance, mean of the $\log_{10}$ transformed reaction times for correct responses);
b) Visual learning and memory: In this task, playing cards are presented on-screen and participants respond 'yes' if the card has appeared in the task before and 'no' if it has not (duration, up to five minutes) (outcome measure = accuracy of performance, arcsine proportion correct);
c) Executive functioning: In this spatial problem solving task, participants are shown a grid of tiles on-screen and using their mouse they must find a hidden pathway on the basis of trial and error feedback (five minutes) (outcome measure = number of errors).

The *CogState* test battery will be located on a dedicated testing portal, linked to the trial website, with participants instructed to complete the cognitive tasks as part of the baseline, post-program (three-months), six and 12-month follow-up assessment. Scores will be compared between the two randomised groups at each time-point.

Adherence: Adherence to CVD treatments will be measured using the Medical Outcomes Study Measures of Patient Adherence Scale. Comprising both general and specific measures, this scale uses self report of adherence to a number of dimensions. The general

**Table 2 Outcome Measures and Assessments**

| | Baseline Assessment at t = 0 months | Post-intervention Assessment at t = 3 months | Follow-up Assessment at t = 6 months | Final Assessment at t = 12 months |
|---|---|---|---|---|
| **Primary Outcome:** | | | | |
| PHQ-9 | + | + | + | + |
| **Secondary Outcomes**: | | | | |
| CogState | + | + | + | + |
| MOS | + | + | + | + |
| **Other Outcomes**: | | | | |
| PSQI | + | + | + | + |
| GAD-7 | + | + | + | + |
| WHODAS | + | + | + | + |
| AUDIT-C | + | + | ... | + |
| BIPQ | + | + | ... | + |
| AABS | + | + | + | + |
| Medication Use | + | + | + | + |
| Employment | + | + | + | + |
| Program satisfaction | ... | + | ... | ... |
| Help-seeking | ... | + | + | + |

Table legend text: PHQ-9 - Patient Health Questionnaire; CogState - series of cognitive tests; MOS - The Medical Outcomes Study Measures of Patient Adherence; PSQI - Pittsburgh Sleep Quality Index; GAD-7 - Generalised Anxiety Disorder Scale; WHODAS - World Health Organisation Disability Assessment Scale; AUDIT-C - Alcohol Use Disorders Identification Test; BIPQ - Brief Illness Perception Questionnaire; AABS - Active Australia Baseline Survey.

measure (five items) evaluates a patient's tendency to adhere to medical recommendations (scored as an average of the five items), while the specific measure (scored as a 0-100 scale) focuses on adherence to medication, exercise, diet and social support (an important additional factor in cardiovascular outcome post event). It has been shown to be sensitive to changes in mood [36]. Changes in mean general and specific adherence behaviour scores will be calculated and compared between the two randomised groups at each time-point.

#### *Other Outcomes*

Other outcomes include: anxiety (using the Generalised Anxiety Disorder Scale; GAD-7) [37], sleep quality (using the Pittsburgh Sleep Quality Index; PSQI) [38], disability (using the World Health Organisation Disability Assessment Scale; WHODAS) [39], illness perception (using the Brief Illness Perception Questionnaire; BIPQ) [40], participation in physical activity (using the Active Australia Baseline Survey; AABS) [41] and alcohol use (using the Alcohol Use Disorders Identification Test - Consumption; AUDIT-C) [42]. Medication use for CVD and depression will also be determined, in addition to workforce and social participation. Satisfaction with treatment and reasons for drop-out will also be measured. Help-seeking using scales measuring actions to overcome depression, preferences for treatment type and expectations of the trial will be measured using previously developed formats [29,43]. User behaviour (e.g. time on site, number of modules completed, length of individual module use and frequency of access) will be tracked for both the *e-couch* and *HealthWatch* programs. In addition, demographic and past medical history data derived from the *45 and Up Study* baseline dataset will be available for analysis.

Refer to Table 2 for a list of all outcomes to be assessed.

### Blinding and allocation concealment

The investigators, analysts, trial manager and all participants will be blinded to treatment allocation for the duration of the trial. Only the internet program system administrator will have access to un-blinded data at the individual level however they will not have any contact with trial participants.

Treatment allocation will be preserved by virtue of the intervention delivery method being web-based, in addition to all of the follow-up assessments. Where participants are unable to complete follow-up assessments online, they will be conducted over the telephone by a Research Assistant who will be blinded to allocation. In the event that treatment allocation is revealed to the Research Assistant during the six-month follow-up assessment, the subsequent 12-month follow-up assessment will be conducted by a secondary Research Assistant, who will also be blinded to allocation.

## Statistical Methods

### Sample Size

We will randomly select 8,000 of those meeting trial eligibility criteria from the *45 and Up Study* to participate. A conservative return rate of 20% is expected from this invitation on the basis of results of previous population-based internet trials [44]. Further assuming that only 40% of those agreeing to potentially participate actually consent after having responded or become un-contactable over a one-month period or currently do not demonstrate depressive symptoms or have access to counselling, this leaves a potential sample of about 640. Taking into account a 20% attrition rate post-randomisation, this will provide a final sample of approximately 510.

### Power Calculation

To detect an effect size of 0.3 standard deviations as the difference between randomised groups in mean change in depressive symptom scores at three-months, with $\alpha$ = 0.05 and $\beta$ = 0.90, the required sample size for the trial is 470.

### Statistical Analyses

The primary a priori outcome is change in depressive symptoms between baseline and three-months as measured by the PHQ-9 score. However, internet trials often have missing data. Analyses of the change in this continuous measure will be undertaken on an intention-to-treat basis, including all participants randomised regardless of treatment actually received or withdrawal from the study. Mixed-model repeated measures (MMRM) analyses will be used since this approach can include participants with missing data and incorporate data from intermediate measurement time points of four and eight weeks. Mixed-models yield unbiased and efficient estimates under MCAR (missing completely at random) and MAR (missing at random) assumptions, which are often reasonable in clinical trials. Additional analyses will explore participant characteristics which moderate outcome and, if appropriate, levels of presenting severity associated with significant improvement. The latter analyses will use a Johnson-Neyman approach [45].

A completers analysis on all participants completing at least 75% of the internet modules and all measures will be undertaken as a secondary analysis. Sensitivity analyses based on multiple imputation and on assuming all drop outs are non-responders (worst case scenario) will be undertaken.

The secondary outcomes of change in the continuous measures of cognitive function and self-reported adherence will be analysed in the same way.

## Ethical Considerations

The trial will be undertaken in compliance with the World Medical Association Declaration of Helsinki (revised version of Seoul, 2008), international standards of Good Clinical Practice (GCP) and the applicable regulatory requirements in Australia. The design and implementation of the trial has been approved by The University of Sydney Human Research Ethics Committee (Reference Number: 06-2009/11800).

The trial is bound by Commonwealth and State privacy legislation and guidelines within Australia, including the Health Records and Information Privacy Act 2002. All research data collected including survey responses, cognitive testing results and website activity data will be identified by the Trial ID numbers only. All files linking participant names and contact information to Trial ID numbers will be stored separately from raw research data. All research data collected will be stored on a secure server at the Brain & Mind Research Institute, The University of Sydney. The server will be password protected and access permitted by authorised research staff only.

A suicide/self-harm risk protocol is in place, which includes a list of emergency sources of help. If participants wish to seek treatment elsewhere, they will be advised to speak to their regular general practitioner or will be provided with information to locate an appropriate general practitioner in their local area. A referral list of appropriate health professionals and services is in place.

# Discussion

This trial will bring together a multidisciplinary perspective from psychiatry, cardiology, psychology, neuropsychology and neurology to study links between depression, CVD and cognitive function using a novel intervention. The trial will be one the first of its kind to evaluate the efficacy of a web-based intervention for depression in people aged 45 years and over with significant depressive symptoms who are also being treated for cardiovascular risk factors. In particular, this trial will target people across the range of CVD, especially those in early stage and those hard to access through usual clinical channels.

The intervention itself (*e-couch*) is evidence-based and can be rolled out in a high fidelity manner on a large scale at low cost. It has the capacity to attract those who don't usually seek health care services and to be delivered in areas with limited access, such as rural and remote regions. Further, this will be the first study to evaluate the effect of treating depression on early cognitive impairment, using a novel internet based assessment. The trial will also assess the effect of intervening on people's mood upon adherence to treatment and lifestyle factors important in secondary cardiovascular disease prevention. It will enable exploration of mechanisms by which these may occur in future studies.

The study addresses an Australian national health priority (ageing well, ageing productively). With health reforms placing preventative services at the centre of health efforts, such a scalable intervention may, if efficacious, have an important role in the amelioration of the burgeoning impact of dementia.

### Abbreviations

AABS: Active Australia Baseline Survey; AUDIT-C: Alcohol Use Disorders Identification Test; BIPQ: Brief Illness Perception Questionnaire; CBT: Cognitive Behavioural Therapy; CREDO: Cardiovascular Risk E-couch Depression Outcome; CVD: Cardiovascular Disease; ECG: Electrocardiogram; GAD-7: Generalised Anxiety Disorder Scale; GCP: Good Clinical Practice; IPT: Interpersonal Psychotherapy; K-10: Kessler Psychological Distress Scale; MAR: Missing At Random; MCAR: Missing Completely At Random; MMRM: Mixed-Model Repeated Measures; MOS: The Medical Outcomes Study Measures of Patient Adherence; OECD: Organisation for Economic Co-operation and Development; PHQ-9: Patient Health Questionnaire; PSQI: Pittsburgh Sleep Quality Index; RCT: Randomised Controlled Trial; WHODAS: World Health Organisation Disability Assessment Scale.

### Acknowledgements

The trial is supported by the Cardiovascular Disease and Depression Strategic Research Program (Award Reference No. G08S 4048) funded by the National Heart Foundation of Australia and *beyondblue: the national depression initiative*.
The 45 and Up Study is managed by The Sax Institute in collaboration with major partner Cancer Council New South Wales; and partners the National Heart Foundation of Australia (NSW Division); NSW Health; *beyondblue: the national depression initiative*; Ageing, Disability and Home Care, Department of Human Services NSW; and UnitingCare Ageing.
The trial protocol was implemented and deployed by Ms Kylie Bennett, e-hub web developer and Mr Anthony Bennett, software engineer, at the Centre for Mental Health Research, at the Australian National University.

### Author details

[1]Brain & Mind Research Institute, The University of Sydney, 100 Mallet Street, Camperdown NSW 2050, Australia. [2]Disciplines of Psychiatry and Sleep Medicine, Sydney Medical School, The University of Sydney NSW 2006, Australia. [3]Centre for Mental Health Research, The Australian National University, Canberra ACT 0200, Australia. [4]The George Institute for Global Health, PO Box M201 Missenden Road, Sydney NSW 2050, Australia.

### Authors' contributions

NLC is the CREDO Trial Manager and is responsible for the day-to-day running of the trial and drafted this manuscript. NG, SLN, HC, BN and IBH jointly developed and wrote the protocol from its inception. NG, SLN, HC, BN and IBH are jointly responsible for the academic oversight of the trial and all authors were involved in revising the manuscript and gave final approval for publication.

### Competing interests

HC is a co-developer of the internet program *e-couch* used in the trial. NG and IBH are members of the Cardiac Depression Collaborative Australia Steering Committee which is partly funded by the National Heart Foundation of Australia. The author's declare that they have no other competing interests.

**Received: 1 December 2010 Accepted: 14 January 2011**
**Published: 14 January 2011**

### References

1. Murray CJL, Lopez AD: *The Global Burden of Disease. A comprehensive assessment of mortality and disability from diseases, injuries, and risk factors in 1990 and projected to 2020* Cambridge, MA, Harvard University Press; 1996.
2. Moussavi S, Chatterji S, Verdes E, Tandon A, Patel V, Ustun B: **Depression, chronic diseases, and decrements in health: results from the World Health Surveys.** *Lancet* 2007, **370(9590)**:851-858.
3. Das-Munshi J, Goldberg D, Bebbington PE, Bhugra DK, Brugha TS, Dewey ME, Jenkins R, Stewart R, Prince M: **Public health significance of mixed anxiety and depression: beyond current classification.** *Br J Psychiatry* 2008, **192(3)**:171-177.
4. Cuijpers P, van Straten A, Smit F, Mihalopoulos C, Beekman A: **Preventing the onset of depressive disorders: a meta-analytic review of psychological interventions.** *Am J Psychiatry* 2008, **165(10)**:1272-1280.
5. Blazer DG, Kessler RC, McGonagle KA, Swartz MS: **The prevalence and distribution of major depression in a national community sample: the National Comorbidity Survey.** *Am J Psychiatry* 1994, **151(7)**:979-986.
6. Bunker SJ, Colquhoun DM, Esler MD, Hickie IB, Hunt D, Jelinek VM, Oldenburg BF, Peach HG, Ruth D, Tennant CC, *et al*: **"Stress" and coronary heart disease: psychosocial risk factors.** *Med J Aust* 2003, **178(6)**:272-276.
7. Nicholson A, Kuper H, Hemingway H: **Depression as an aetiologic and prognostic factor in coronary heart disease: a meta-analysis of 6362 events among 146 538 participants in 54 observational studies.** *Eur Heart J* 2006, **27(23)**:2763-2774.
8. Berkman LF, Blumenthal J, Burg M, Carney RM, Catellier D, Cowan MJ, Czajkowski SM, DeBusk R, Hosking J, Jaffe A, *et al*: **Effects of treating depression and low perceived social support on clinical events after myocardial infarction: the Enhancing Recovery in Coronary Heart Disease Patients (ENRICHD) Randomized Trial.** *JAMA* 2003, **289(23)**:3106-3116.
9. Glassman AH, O'Connor CM, Califf RM, Swedberg K, Schwartz P, Bigger JT Jr, Krishnan KR, van Zyl LT, Swenson JR, Finkel MS, *et al*: **Sertraline treatment of major depression in patients with acute MI or unstable angina.[Erratum appears in JAMA 2002 Oct 9;288(14):1720].** *JAMA* 2002, **288(6)**:701-709.
10. van Melle JP, de Jonge P, Honig A, Schene AH, Kuyper AM, Crijns HJ, Schins A, Tulner D, van den Berg MP, Ormel J, *et al*: **Effects of antidepressant treatment following myocardial infarction.** *Br J Psychiatry* 2007, **190**:460-466.
11. Lesperance F, Frasure-Smith N, Koszycki D, Laliberte MA, van Zyl LT, Baker B, Swenson JR, Ghatavi K, Abramson BL, Dorian P, *et al*: **Effects of citalopram and interpersonal psychotherapy on depression in patients with coronary artery disease: the Canadian Cardiac Randomized Evaluation of Antidepressant and Psychotherapy Efficacy (CREATE) trial.** *JAMA* 2007, **297(4)**:367-379, [Erratum appears in JAMA. 2007 Jul 4;298(1):40].
12. Carney RM, Freedland KE, Carney RM, Freedland KE: **Depression and coronary heart disease: more pieces of the puzzle.** *Am J Psychiatry* 2007, **164(9)**:1307-1309.
13. Raynor D, Wing R, Phelan S: **Depression and adherence to medical advice.** In *Depression and Physical Illness.* Edited by: Steptoe A. Cambridge: Cambridge University Press; 2007:369-396.
14. DiMatteo MR, Lepper HS, Croghan TW: **Depression is a risk factor for noncompliance with medical treatment: meta-analysis of the effects of anxiety and depression on patient adherence.** *Arch Intern Med* 2000, **160(14)**:2101-2107.
15. Naismith SL, Hickie IB, Turner K, Little CL, Winter V, Ward PB, Wilhelm K, Mitchell P, Parker G: **Neuropsychological Performance in Patients With Depression is Associated With Clinical, Etiological and Genetic Risk Factors.** *J Clin Exp Neuropsychol* 2003, **25(6)**:866-877.
16. Naismith SL, Longley WA, Scott EM, Hickie IB: **Disability in major depression related to self-rated and objectively-measured cognitive deficits: a preliminary study.** *BMC Psychiatry* 2007, **7**:32.
17. Kohler S, Thomas AJ, Barnett NA, O'Brien JT: **The pattern and course of cognitive impairment in late-life depression.** *Psychol Med* 2010, **40(4)**:591-602.
18. Naismith SL, Glozier N, Burke D, Carter PE, Scott E, Hickie IB: **Early intervention for cognitive decline: Is there a role for multiple medical or behavioural interventions?** *Early Interv Psychiatry* 2009, **3(1)**:19-27.
19. Mackinnon A, Griffiths KM, Christensen H: **Comparative randomised trial of online cognitive-behavioural therapy and an information website for depression: 12-month outcomes.** *Br J Psychiatry* 2008, **192(2)**:130-134.
20. Ruggiero KJ, Resnick HS, Acierno R, Coffey SF, Carpenter MJ, Ruscio AM, Stephens RS, Kilpatrick DG, Stasiewicz PR, Roffman RA, *et al*: **Internet-based intervention for mental health and substance use problems in disaster-affected populations: a pilot feasibility study.** *Behav Ther* 2006, **37(2)**:190-205.
21. Griffiths KM, Christensen H: **Review of randomised controlled trials of internet interventions for mental disorders and related conditions.** *Clin Psychol* 2006, **10(1)**:16-29.

22. Spek V, Cuijpers P, Nyklicek I, Smits N, Riper H, Keyzer J, Pop V: **One-year follow-up results of a randomized controlled clinical trial on Internet-based cognitive behavioural therapy for subthreshold depression in people over 50 years.** *Psychol Med* 2008, **38(5)**:635-639.
23. van Bastelaar KM, Pouwer F, Cuijpers P, Twisk JW, Snoek FJ: **Web-based cognitive behavioural therapy (W-CBT) for diabetes patients with co-morbid depression: design of a randomised controlled trial.** *BMC Psychiatry* 2008, **8**:9.
24. Banks E, Redman S, Jorm L, Armstrong B, Bauman A, Beard J, Beral V, Byles J, Corbett S, Cumming R, *et al*: **Cohort profile: the 45 and up study.** *Int J Epidemiol* 2008, **37(5)**:941-947.
25. Mealing NM, Banks E, Jorm LR, Steel DG, Clements MS, Rogers KD: **Investigation of relative risk estimates from studies of the same population with contrasting response rates and designs.** *BMC Med Res Methodol* 2010, **10**:26.
26. Christensen H, Griffiths MK, Korten A: **Web-based Cognitive Behavior Therapy: Analysis of Site Usage and Changes in Depression and Anxiety Scores.** *J Med Internet Res* 2002, **4(1)**:e3.
27. Jorm AF, Christensen H, Griffiths KM, Rodgers B: **Effectiveness of complementary and self-help treatments for depression.** *Med J Aust* 2002, **176(Suppl)**:S84-96.
28. Griffiths KM, Christensen H: **Internet-based mental health programs: a powerful tool in the rural medical kit.** *Aust J Rural Health* 2007, **15(2)**:81-87.
29. Christensen H, Griffiths KM, Jorm AF: **Delivering interventions for depression by using the internet: randomised controlled trial.** *BMJ* 2004, **328(7434)**:265.
30. Lowe B, Spitzer RL, Grafe K, Kroenke K, Quenter A, Zipfel S, Buchholz C, Witte S, Herzog W: **Comparative validity of three screening questionnaires for DSM-IV depressive disorders and physicians' diagnoses.** *J Affect Disord* 2004, **78(2)**:131-140.
31. Lowe B, Unutzer J, Callahan CM, Perkins AJ, Kroenke K: **Monitoring depression treatment outcomes with the patient health questionnaire-9.** *Med Care* 2004, **42(12)**:1194-1201.
32. Weaver Cargin J, Collie A, Masters C, Maruff P: **The nature of cognitive complaints in healthy older adults with and without objective memory decline.** *J Clin Exp Neuropsychol* 2008, **30(2)**:245-257.
33. Falleti MG, Maruff P, Collie A, Darby DG: **Practice effects associated with the repeated assessment of cognitive function using the CogState battery at 10-minute, one week and one month test-retest intervals.** *J Clin Exp Neuropsychol* 2006, **28(7)**:1095-1112.
34. Maruff P, Collie A, Darby D, Weaver-Cargin J, Masters C, Currie J: **Subtle memory decline over 12 months in mild cognitive impairment.** *Dement Geriatr Cogn Disord* 2004, **18(3-4)**:342-348.
35. Maruff P, Thomas E, Cysique L, Brew B, Collie A, Snyder P, Pietrzak RH: **Validity of the CogState brief battery: relationship to standardized tests and sensitivity to cognitive impairment in mild traumatic brain injury, schizophrenia, and AIDS dementia complex.** *Arch Clin Neuropsychol* 2009, **24(2)**:165-178.
36. Ziegelstein RC, Fauerbach JA, Stevens SS, Romanelli J, Richter DP, Bush DE: **Patients with depression are less likely to follow recommendations to reduce cardiac risk during recovery from a myocardial infarction.** *Arch Intern Med* 2000, **160(12)**:1818-1823.
37. Spitzer RL, Kroenke K, Williams JB, Lowe B: **A brief measure for assessing generalized anxiety disorder: the GAD-7.** *Arch Intern Med* 2006, **166(10)**:1092-1097.
38. Buysse DJ, Reynolds CF, Monk TH, Berman SR, Kupfer DJ: **The Pittsburgh Sleep Quality Index: a new instrument for psychiatric practice and research.** *Psychiatry Res* 1989, **28(2)**:193-213.
39. Kim JM, Stewart R, Glozier N, Prince M, Kim SW, Yang SJ, Shin IS, Yoon JS: **Physical health, depression and cognitive function as correlates of disability in an older Korean population.** *Int J Geriatr Psychiatry* 2005, **20(2)**:160-167.
40. Broadbent E, Petrie KJ, Main J, Weinman J: **The brief illness perception questionnaire.** *J Psychosom Res* 2006, **60(6)**:631-637.
41. Brown WJ, Burton NW, Marshall AL, Miller YD: **Reliability and validity of a modified self-administered version of the Active Australia physical activity survey in a sample of mid-age women.** *Aust NZ J Public Health* 2008, **32(6)**:535-541.
42. Bush K, Kivlahan DR, McDonell MB, Fihn SD, Bradley KA: **The AUDIT alcohol consumption questions (AUDIT-C): an effective brief screening test for problem drinking. Ambulatory Care Quality Improvement Project (ACQUIP). Alcohol Use Disorders Identification Test.** *Arch Intern Med* 1998, **158(16)**:1789-1795.
43. Christensen H, Leach LS, Barney L, Mackinnon AJ, Griffiths KM: **The effect of web based depression interventions on self reported help seeking: randomised controlled trial [ISRCTN77824516].** *BMC Psychiatry* 2006, **6**:13.
44. Christensen H, Griffiths KM, Farrer L: **Adherence in internet interventions for anxiety and depression.** *J Med Internet Res* 2009, **11(2)**:e13.
45. Johnson PO: **Tests of certain linear hypotheses and their application to some educational problems.** In *Statistical Research Memoirs. Volume 1.* Edited by: Neyman J, Pearson ES. London: University College; 1936:57-93.

### Pre-publication history

The pre-publication history for this paper can be accessed here:
http://www.biomedcentral.com/1471-244X/11/10/prepub

doi:10.1186/1471-244X-11-10
**Cite this article as:** Cockayne *et al.*: Internet-based treatment for older adults with depression and co-morbid cardiovascular disease: protocol for a randomised, double-blind, placebo controlled trial. *BMC Psychiatry* 2011 **11**:10.

**Submit your next manuscript to BioMed Central and take full advantage of:**

- **Convenient online submission**
- **Thorough peer review**
- **No space constraints or color figure charges**
- **Immediate publication on acceptance**
- **Inclusion in PubMed, CAS, Scopus and Google Scholar**
- **Research which is freely available for redistribution**

Submit your manuscript at
www.biomedcentral.com/submit

BioMed Central
